# Understanding the mechanisms through which women's group community participatory intervention improved maternal health outcomes in rural Malawi: was the use of contraceptives the pathway?

**DOI:** 10.3402/gha.v9.30496

**Published:** 2016-04-19

**Authors:** Collins O. F. Zamawe, Chrispin Mandiwa

**Affiliations:** 1 Parent and Child Health Initiative, Research Centre, Lilongwe, Malawi; 2 Ministry of Health, Lilongwe, Malawi; 3 School of Public Health, College of Medicine, University of Malawi, Lilongwe, Malawi

**Keywords:** community-based intervention, women's health, community health promotion, contraception

## Abstract

**Background:**

Women's group intervention is a community based initiative through which rural women form groups, meet regularly to discuss maternal health issues affecting them, and come up with locally available solutions. This intervention has been associated with reduced maternal and neonatal mortality in limited resource settings. Nevertheless, the mechanisms through which women's groups influence maternal health outcomes are uncertain. Because contraception reduces the risk of maternal mortality and women's groups also tackled this issue, we speculated that contraceptive use might be the pathway. Consequently, this study investigated whether participation in women's groups was associated with contraceptive use in Malawi.

**Design:**

We examined the use of contraceptives between women who participated in women's groups and those who did not through a community-based cross-sectional study in Mchinji, Malawi. The study involved 3,435 women of reproductive age (15–49 years) who were recruited using a multistage sampling approach. Members (treated) and non-members (control) of women's groups were matched on observed covariates using propensity scores and the counterfactual for the treated individuals was estimated.

**Results:**

Crude analysis revealed that women's groups improved uptake of contraceptives by 26% (odds ratio (OR)=1.26; 95% confidence interval (CI)=1.03–1.56; *p*=0.024). However, using the matched data, uptake of contraceptives was almost the same among members and non-members of women's groups. More precisely, the likelihood of using contraceptives was not significantly different between the members and non-members of women's groups (OR=1.00; 95% CI=0.81–1.24; *p*=0.991).

**Conclusions:**

There is insufficient evidence of an association between participation in women's groups and contraceptive use among rural Malawian women. The implication is that contraception was not the mechanism through which women's groups contributed to reduced maternal mortality in Malawi. Because the effects of community interventions are usually comprehensive and sometimes difficult to demonstrate, ethnographic studies should be considered in the evaluation of women's groups and other related interventions.

## Paper context

Women's group intervention contributes to reduced maternal mortality. However, its mechanism of action remains unknown. Because contraception reduces the risk of maternal mortality and women's groups addressed this issue, we hypothesised that improved use of contraceptives was the pathway. The findings demonstrate that there is unsatisfactory evidence of an association between women's groups and contraceptive use. In future, the mechanisms through which women's groups reduce maternal mortality should be explored through in-depth qualitative studies.

## Introduction

Even though the maternal mortality ratio (MMR) has gone down by about 45% worldwide since 1990, many women continue to die each year due to preventable pregnancy-related complications (1). Most of these deaths occur in low- and middle-income countries (LMICs) (1). A closer look at the global maternal mortality trends divulges more socio-economic-based disparities between regions, nations, and districts/provinces (2). In Africa, for example, MMR in sub-Saharan Africa is over seven times higher than in Northern Africa (3). Likewise, women in Southern Asia are about six times more likely to die of pregnancy-related complications than their counterparts in East Asia (3). Similar differences also exist within countries between rich and poor women (2, 4).

Effective interventions are available to prevent or manage almost all pregnancy-related complications, but they do not adequately reach those who need them most (3, 4). Contraception, antenatal care, facility delivery, and postnatal care are some of the known interventions that have the potential to avert most maternal deaths (3, 5, 6). However, access to these services seems to favour those at lower risk. For instance, although MMR is highest in sub-Saharan Africa, women in this region have lower access to maternal healthcare services (2, 7, 8). Even at a country level, women who are at a greater risk of maternal death (i.e. the poor, uneducated, and rural dwellers) have relatively low access to health services (2, 3, 6, 9).

Lately, there has been increased focus on community-based interventions for maternal health, especially in LMICs (10–12). This is probably due to the persistent exclusion of the poor from the mainstream healthcare system. One of the promising community interventions for maternal health is the creation of women's groups in rural areas with low access to health services (8). This is an intervention through which rural women form groups in their own villages, meet regularly to discuss maternal health issues affecting them, and come up with locally available solutions (13–15). So far, women's groups have been implemented and evaluated in Nepal, India, Bangladesh, Bolivia, and Malawi (16–20). Overall, this intervention is associated with reduced maternal and neonatal mortality in LMICs (21).

Although women's group intervention is known to contribute to improved maternal health outcomes, its mechanisms of action are uncertain. For instance, while acknowledging the role of women's groups in maternal health, the World Health Organization observed that there is insufficient evidence that the intervention improves 1) institutional delivery, 2) birth with a skilled attendant, 3) number of antenatal care visits, and 4) uptake of postnatal care (8). Comparable observations were also reported by a meta-analysis of seven randomised controlled trials that examined the effectiveness of women's groups (21).

Contraception has long been associated with reduced risk of maternal mortality (3, 5). Because women's groups to a certain extent have tackled this issue, contraceptive use is a potential pathway through which the groups could have improved maternal health outcomes in Malawi. Nevertheless, the use of contraceptives has not been thoroughly examined vis-à-vis women's groups. For that reason, the objective of this study was to investigate if participation in women's groups was associated with contraceptive use. The ultimate goal was to understand if contraception is the missing link between women's groups and maternal health.

## Methods

### Women's groups in Malawi

In Malawi, women's groups were implemented in Mchinji District from 2005 to 2010. The intervention was supported by the MaiMwana Project, Ministry of Health, and the University College London's Institute for Global Health. The overall goal of the women's group intervention was to empower community members in rural Malawi (especially women) to take control of the maternal and child health problems that affect them (13). This intervention was implemented within a factorial randomised controlled trial, which was designed to evaluate its impact. Mchinji District was divided into 48 identical clusters, and 24 of these received women's group intervention (13, 22). Community members in the selected clusters were mobilised, and 310 villages chose to establish 207 women's groups. Membership criteria were group-specific, but Phases 1 and 2 (see below) were restricted to women.

Just like in Nepal and Bolivia, women's groups in Malawi have adopted a participatory learning and action cycle (16, 18). This model comprised four phases and a series of 20 monthly meetings. In Phase 1, the groups identified and prioritised maternal and neonatal health issues affecting women in their respective communities. In Phase 2, they identified feasible strategies for addressing the listed priority problems and went on to implement the strategies in Phase 3. Phase 4 involved evaluating the first three phases. Local facilitators were identified and trained to support the groups in their respective communities.

It should be emphasised that the women's group activities were driven by maternal and neonatal priority issues that women identified in their individual groups. Therefore, family planning was not the main focus of the women's groups, but the topic was addressed by some groups within the broader context of maternal health (i.e. when groups identified high parity or closely spaced pregnancies as a factor contributing to maternal mortality). Evaluation of the women's groups in Malawi demonstrated that the intervention effectively improved both maternal and neonatal mortality (22). Detailed information about women's groups in Malawi has been published elsewhere (13, 19, 22).

### Study design and setting

We examined the use of contraceptives between women who participated in women's groups and those who did not through a community-based cross-sectional study. The study was conducted in Mchinji, a typical rural district in the Central Region of Malawi. The district has nine traditional authorities (communities). It has a population of about 500,000 and covers an area of approximately 3,500 square kilometres (13).

### Population and data collection

The target population of the study was women of reproductive age (15–49 years). To be eligible, women were supposed to be permanent residents of Mchinji and must have stayed in the district during the implementation period of women's groups. In total, 3,825 women participated in this study. However, some cases (*n*=386) were discarded due to incomplete data (23). Thus, the final sample size was 3,435. Respondents were identified through a multistage cluster sampling method. The first stage involved selecting clusters (24 out of 48) using a simple random sampling method (24). In the second stage, 160 households were selected in each cluster through a systematic random sampling approach (25). One eligible and available woman was interviewed per each selected household. In case of ties, priority was given to the main woman (mother) or a simple random sampling method was used. Data were collected using structured questionnaires. The questionnaires captured different types of information from the respondents, including data on demographic factors, use of maternal healthcare services, use of contraceptives, and participation in women's groups.

### Study variables

The use of contraceptives was the outcome variable of this study. Respondents were asked if they had ever used any method of contraception. Those who answered yes had to specify the method or methods used. The main independent variable was participation in women's groups. This was defined as direct participation (membership) or secondary access to women's group materials or information from other people. Covariates for this study were chosen theoretically based on previous literature on contraceptive use in LMICs (26, 27). Therefore, the following variables were considered relevant: age of respondents, marital status, number of children/pregnancies, occupation, highest education level, place of delivery, uptake of antenatal and postnatal care, family planning decision maker, religion, and exposure to other similar interventions.

### Data analysis

We regarded women's group membership as treatment. Thus, women who participated in women's groups (treated) were compared against those who did not participate (control). Because the treatment assignment was not random, selection bias was evident (28). As a result, we used a propensity score matching (PSM) method to balance measured covariates across the groups (treatment and control) and then examine the treatment effects of women's groups on contraceptive use. Covariates were considered balanced if the standardised differences were below 10% (28, 29). The treated group was matched with the control on all the study covariates (see above) except family planning decision maker, because it was likely to be influenced by the intervention. We used kernel matching (type=epan and bandwidth=0.06) because it provided the greatest amount of bias reduction. In addition, we also computed descriptive statistics. All statistical tests were deemed significant if alpha (α) was <0.05.

### Ethics statement

This study was part of the Phukusi la Moyo research project, which was duly approved by the National Health Sciences Research Committee (NHSRC) in Malawi. In addition, written informed consent was obtained from all respondents. Parents/guardians provided additional consent for participants who were below the age of 18 years.

## Results

Table 1 presents the characteristics of the respondents and factors associated with the use of contraceptives in Mchinji. Of the 3,435 respondents who were included in the analysis, 1,098 (32.0%) participated in the women's groups. In general, most of the respondents (47.3%) were between the ages of 20 and 29 years. In addition, almost all the respondents (97.1%) were either married or formerly married. A considerable proportion (84.5%) of the participants had acquired at least a basic education and were subsistence farmers (74.5%).

**Table 1:** Descriptive statistics and factors associated with the use of contraceptives (unmatched data)

			Used contraceptives				
				
			No		Yes		
					
Variable	*n*	%	*n*	%	*n*	%	*p*
Age (years)							
15–19	221	6.4	106	48	115	52	<0.001*
20–29	1,624	47.3	207	12.8	1,417	87.2	
30–39	1,183	34.4	135	11.4	1,048	88.6	
40–49	407	11.9	82	20.2	325	79.8	
Education							
None	533	15.5	108	20.3	425	79.7	0.001*
Primary school	2,451	71.4	345	14.1	2,451	85.9	
Secondary school	451	13.1	77	17.1	374	82.9	
Marital status							
Never married	100	2.9	42	42	58	58	<0.001*
Currently married	3,094	90.1	427	13.8	2,667	86.2	
Formerly married	241	7.0	61	25.3	180	74.7	
Occupation							
Farmer	2,559	74.5	384	15	2,175	−85	<0.001*
Self-employed	515	15	46	17.2	222	82.8	
Casual worker	268	7.8	70	13.6	445	86.4	
Other	93	2.7	30	32.3	63	67.7	
Women's group member							
No	2,337	68	383	16.4	1,954	83.6	<0.023*
Yes	1,098	32	147	13.4	951	86.6	
Religion							
Christian	2,755	80.2	504	18.3	2,251	81.7	0.509**
Muslim or other	680	19.8	117	17.2	563	82.8	
Utilised antenatal care							
No	54	1.6	27	50	27	50	<0.001*
Yes	3,381	98.4	503	14.9	2,878	85.1	
Exposure to other programme							
No	1,346	39.2	264	19.6	1,082	80.4	<0.001*
Yes	2,089	60.8	266	12.7	1,823	87.3	
Facility delivery							
No	266	7.7	82	30.8	184	69.2	<0.001*
Yes	3,169	92.3	448	14.1	2,721	2,905	
Utilised postnatal care							
No	471	13.7	125	26.5	346	73.5	<0.001*
Yes	2,964	86.3	405	13.7	2,559	86.3	
Family planning decision							
Woman	1,698	57.3	46	2.7	1,652	97.3	0.554**
Husband	1,268	42.7	39	3.1	1,229	96.9	
Total	3,435	100	530	15.4	2,905	84.6	

* Significant (p≤0.05)

** Non-significant (p>0.05).

**Fig. 1:**
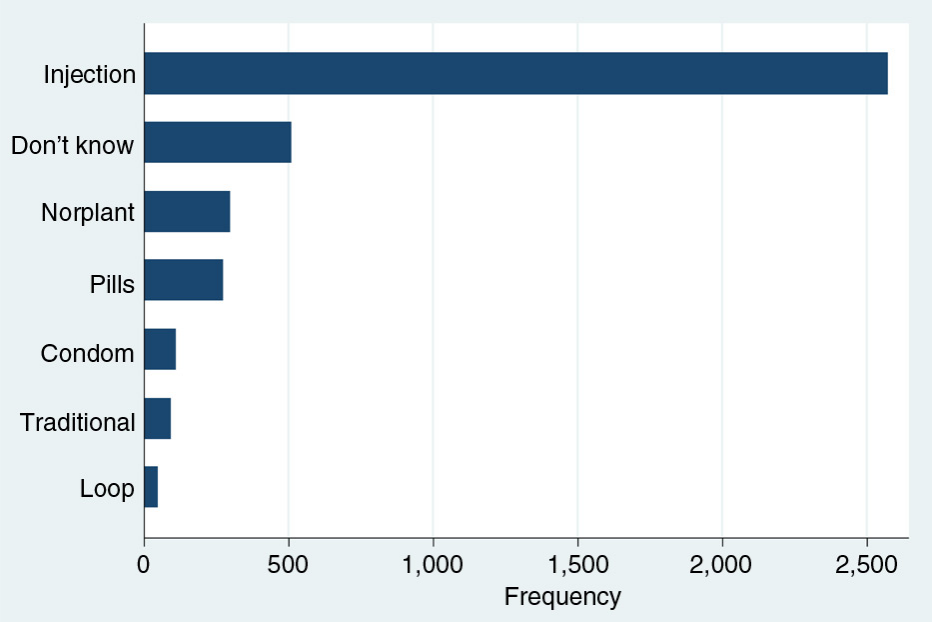
Methods of contraception ever used by women in Mchinji (*n*=2,905).

Overall, the use of contraceptives among women was very high in Mchinji. Of the 3,435 participants, 2,905 (84.6%) were using or had ever used contraceptives. Injection was the most common method of contraception (Fig. 1) and short-term methods were more popular (88.0%) compared to long-term ones (12.0%). In addition, more decisions regarding contraception were made by women (57.3%) than men (42.7%), though this was not significantly associated with the use of contraceptives (*p*=0.554). Most of the characteristics of the respondents were significantly associated with uptake of contraceptives. Particularly, participation in women's groups was related to the use of contraceptives (*p*=0.023).

**Fig. 2:**
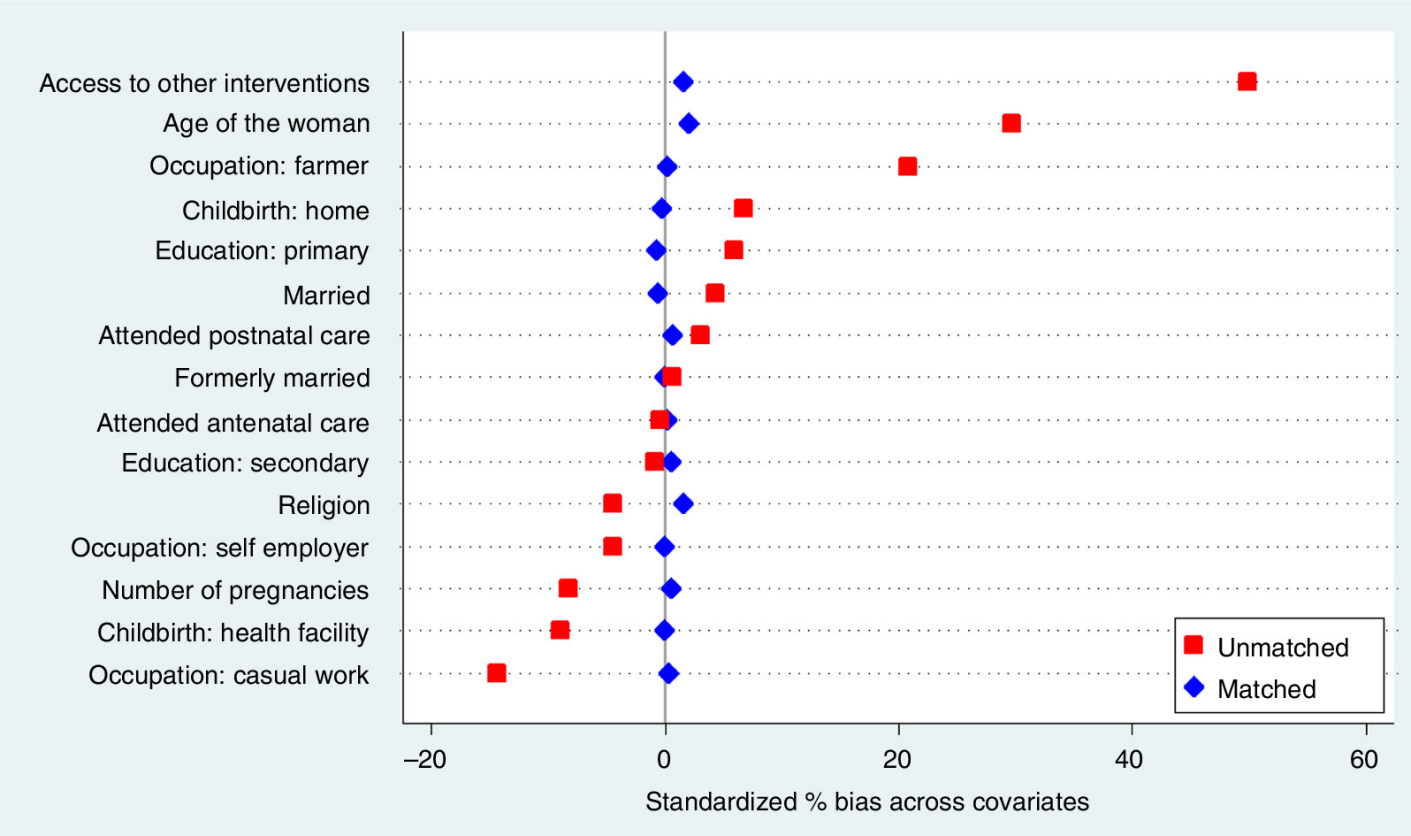
Standardise differences across covariates before and after propensity score matching.

We also specifically examined the use of contraceptives among women's group members and non-members. Initial analysis revealed that women's groups improved uptake of contraceptives by 26% (odds ratio (OR)=1.26; 95% confidence interval (CI)=1.03–1.56; *p*=0.024). Nevertheless, apart from participation in women's groups, respondents were also different in their baseline characteristics (30). Therefore, we used a PSM method to balance observed covariates across members (treatment) and non-members (control) of the women's groups (29) and then estimated the counterfactual for the members (28). PSM significantly reduced the standardised differences across all covariates in our sample (Fig. 2).

**Fig. 3:**
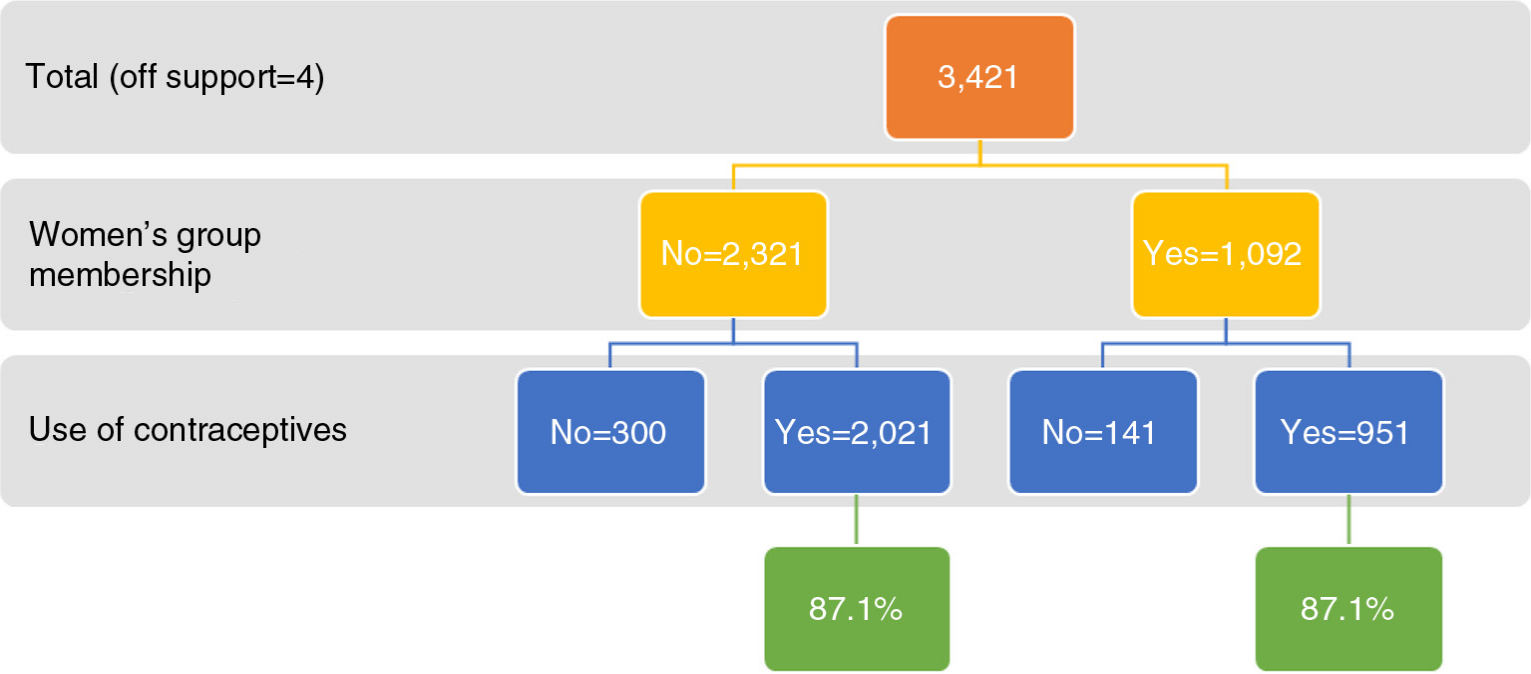
The use of contraceptives between women's group members and non-members in Malawi (matched data).

Using the matched data, we had another look at the effect of women's groups on the use of contraceptives. Uptake of contraceptives was almost the same among members and non-members of women's groups (Fig. 3). More precisely, the likelihood of using contraceptives was not significantly different between the two groups (OR=1.00; 95% CI=0.81–1.24; *p*=0.991).

## Discussion

The role of women's group interventions in maternal health in LMICs cannot be overstated. In this study, the effect of the intervention on the use of contraceptives was assessed. We have established that the use of contraceptives among members and non-members of women's groups was not substantially different. In other words, women's groups did not significantly influence uptake of contraceptives among rural women in Malawi. Consequently, this outcome rules out contraception as a possible mechanism through which women's groups contributed to reduced maternal mortality in Malawi.

The finding that contraception is not the link between women's groups and maternal health is consistent with existing evidence. For instance, studies have shown that women's groups’ mechanism of action is not related to utilisation of maternal healthcare services (8, 21). Existing evidence indicates that in one way or another antenatal care, delivery with a skilled birth attendant, childbirth at a health facility, postnatal care, and contraception are associated with improved maternal health outcomes in LMICs (5, 6, 31). As a result, these are potential pathways through which women's groups could influence maternal health outcomes. Of these, only contraception had not been systematically examined in relation to women's groups. Therefore, this study bridges an important gap in the literature of women's groups and maternal health.

With this in mind, how do women's groups improve maternal health outcomes? One of the known impacts of the women's group intervention is that it improves hygiene during childbirth (21). This is because the intervention provides a platform where women share important health information about hygiene, malaria, and HIV, among others (18, 21, 22). Therefore, it is possible that women's groups contribute to reduced maternal mortality by minimising the risk of puerperal sepsis (21). This hypothesis requires thorough exploration.

In addition, it is usually challenging to establish the actual pathways of outcomes in multifaceted community-based interventions. Therefore, the link between women's groups and maternal health is probably neither straightforward, simple, nor empirically quantifiable (8). For that reason, it is important to examine the qualitative outcomes of the intervention. This may possibly enhance our understanding of the role of women's groups in maternal health.

As already elucidated, the main focus of women's group intervention in Malawi was maternal and neonatal health problems (13). This suggests that the intervention did not adequately address issues of contraception. Because of this, our results do not necessarily put forward the concept that women's groups cannot effectively improve the use of contraceptives among rural women. The findings simply indicate that contraception was not the channel through which women's groups influenced maternal mortality in rural Malawi.

The findings of this study should be considered in light of the following limitations and strengths. First, data on both outcome and independent variables were self-reported. To the extent that the participants misreported, our results may be biased. Moreover, due to the cross-sectional nature, no temporal linkages can be made. Moreover, sampling of participants was not completely random; hence, the results may not be generalised. On the other hand, the use of PSM greatly reduced selection bias, which is a major issue in observational studies. Additionally, our sample size was relatively large, which implies more statistical power.

## Conclusions

There is insufficient evidence of an association between participation in women's groups and contraceptive use among rural women in Malawi. In other words, participation in women's group intervention did not influence uptake of contraceptives. The implication of this finding is that contraception may not be the pathway through which women's groups contributed to reduced maternal mortality in Malawi. Thus, the mechanisms of action for women's groups remain ambiguous. Given that the effects of community interventions are usually comprehensive and sometimes difficult to demonstrate, rigorous ethnographic studies should be part of the evaluation of women's groups. This would deepen our understanding of the link between women's groups and maternal health beyond the numbers or statistics.
